# More Is Not Enough: A Deeper Understanding of the COVID-19 Impacts on Healthcare, Energy and Environment Is Crucial

**DOI:** 10.3390/ijerph18020684

**Published:** 2021-01-14

**Authors:** Peng Jiang, Jiří Jaromír Klemeš, Yee Van Fan, Xiuju Fu, Yong Mong Bee

**Affiliations:** 1 Department of Systems Science, Institute of High Performance Computing (IHPC), Agency for Science, Technology and Research (A*STAR), Singapore 138632, Singapore; Jiang_Peng@ihpc.a-star.edu.sg (P.J.); fuxj@ihpc.a-star.edu.sg (X.F.); 2 Sustainable Process Integration Laboratory—SPIL, NETME Centre, Faculty of Mechanical Engineering, Brno University of Technology—VUT Brno, Technická 2896/2, 616 69 Brno, Czech Republic; fan@fme.vutbr.cz; 3 Department of Endocrinology, Singapore General Hospital (SGH), Singapore 169608, Singapore; bee.yong.mong@singhealth.com.sg

**Keywords:** COVID-19 impacts, co-epidemics, healthcare–energy–environment nexus, climate footprint, sustainable development, interdisciplinary analysis

## Abstract

The coronavirus disease 2019 (COVID-19) pandemic has magnified the insufficient readiness of humans in dealing with such an unexpected occurrence. During the pandemic, sustainable development goals have been hindered severely. Various observations and lessons have been highlighted to emphasise local impacts on a single region or single sector, whilst the holistic and coupling impacts are rarely investigated. This study overviews the structural changes and spatial heterogeneities of changes in healthcare, energy and environment, and offers perspectives for the in-depth understanding of the COVID-19 impacts on the three sectors, in particular the cross-sections of them. Practical observations are summarised through the broad overview. A novel concept of the healthcare–energy–environment nexus under climate change constraints is proposed and discussed, to illustrate the relationships amongst the three sectors and further analyse the dynamics of the attention to healthcare, energy and environment in view of decision-makers. The society is still on the way to understanding the impacts of the whole episode of COVID-19 on healthcare, energy, environment and beyond. The raised nexus thinking could contribute to understanding the complicated COVID-19 impacts and guiding sustainable future planning.

## 1. Introduction

**Figure 1:**
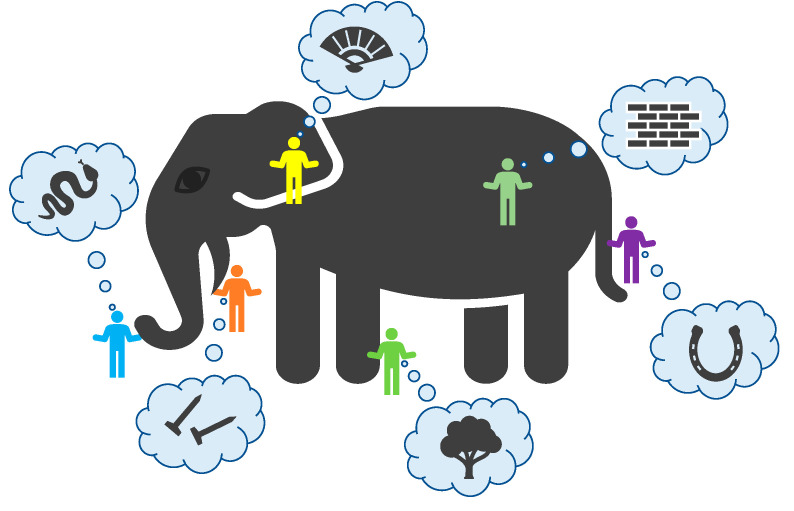
The parable of the blind men and the elephant (amended from a Hindoo parable)—It feels like a snake; it feels like a spear; it feels like a fan; it feels like a tree; it feels like a wall; no, it feels like a rope!. The picture was amended, developed and drawn by the authors.

Since coronavirus disease 2019 (COVID-19) was first recognised and reported, over 80 million people, from 222 countries, have been infected with coronavirus, and around 1.8 million of them have lost their lives within about a year [1]. The sustainable development goals have been hindered by COVID-19 severely [2]. Although it is understandable, and there is no doubt that profound influences have been created and observed in numerous aspects [3], this study focuses on three important things—namely, healthcare, energy and environment—that are being coupled more closely than ever during the COVID-19 pandemic. Facing the uncertainties caused by COVID-19 in the past 12 months, society is still on the way to understanding the impacts of the whole episode of the COVID-19 on healthcare, energy, environment and beyond. As mentioned in the famous proverb “the blind men and the elephant” [4] based on a Hindoo Fable (See Figure 1), people contribute limited knowledge and experience to the understanding of an unfamiliar world. For the current situation, a more in-depth understanding is crucial based on group wisdom and prompt information sharing.

**Figure 2:**
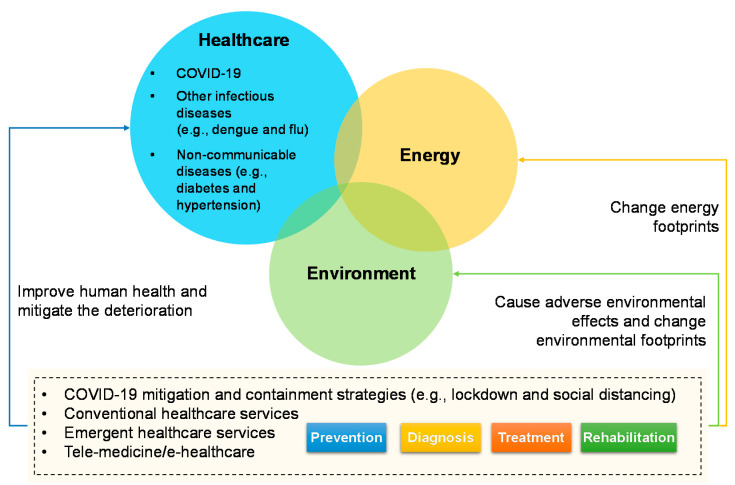
The healthcare–energy–environment system during the COVID-19 pandemic.

In the healthcare–energy–environment system in Figure 2, both conventional and emergent healthcare services, including e-healthcare and telemedicine [5], consume energy. Energy is key to healthcare services, which has played a fundamental role in the emergency response of the COVID-19 pandemic [6]. During the COVID-19 response, the energy insecurity and city environment have plagued primary healthcare systems, especially in the middle- and low-income countries [7]. Environmental factors have been broadly thought to be drivers for different infectious diseases, both in the land [8] and in the ocean [9]. Many epidemic and pandemic events were caused by the contact between environmental media and humans [10], which constitutes a complex nexus between the natural environment and human society [11]. In addition, both the healthcare and energy sectors would cause adverse environmental effects. To this end, COVID-19 should not be an isolated public health incident, either in terms of its occurrence or from its impact perspective. Sound healthcare systems stressed the balance between the health of human beings and the disruptions of ecology that we engender [12]. The on-going COVID-19 demonstrated this relationship where any disruption of the human–ecological balance implicates human health [12]. Energy and environment are changing the vulnerability of healthcare to disease prevention, including COVID-19, other infectious diseases and non-communicable diseases. For example, severe air pollution was found to be linked with higher mortality of patients when infected by the coronavirus [13]. Indoor air quality which causes respiratory issues has become an issue during the lockdown, relating to the ventilation and intensive use of cleaning products [14]. Unequal access to environmental and sanitation resources varied the capability to perform preventative measures. There are also studies suggested that the COVID-19 outbreak stems from environmental degradation, related to the exploitation of natural resources, emerging from the infectious diseases of zoonotic origin [15]. The relationship is not one-way, but a network or coupling system. COVID-19 that is viewed as the current healthcare emergency in most countries is also having indirect impacts on other diseases (e.g., treatment priority), energy consumption (e.g., caused by prevention strategies), and environmental footprint (e.g., changed by resources consumption).

COVID-19 challenged healthcare systems, changed population lifestyles, reshaped energy consumption and living environment in society, and further triggered a series of chain reactions in the healthcare–energy–environment system. Various observations and quick lessons were published, via the fast track, to highlight the local impact on a single region or single sector, e.g., the healthcare impact in Latin America [16] and India [17], the short-run impact on the electricity sector in the United States (US) [18], the energy grid dynamics in Europe [19], the impact on air quality in Brazil [20], and the assessment of the impact on the environment in China [21]. Although the aforementioned studies did not pay attention to broader situations, they did contribute to understanding the specific impacts of COVID-19, which lay the foundation for further systematic integration. Other relevant studies are overviewed and discussed in Section 2 to Section 4. The more recent overview papers focused on multiple sectors independently. Mentioning at least a few: Eroğlu [22] discussed the COVID-19 effects on the environment and renewable energy separately. Chakraborty and Maity [23] briefly described the COVID-19 impact on the global environment and the society in terms of economy and health. Gillingham et al. [24] made a commentary on both the short-term and long-term effects on energy and the environment. Nicola et al. [3] summarised the COVID-19 impacts on different socio-economic sectors respectively, including the petroleum and oil, agriculture, manufacturing, education, finance, healthcare, tourism and food sectors. Mofijur et al. [25] offered an overview of the COVID-19 impacts on energy, environment and socio-economic sectors separately. Elavarasan and Pugazhendhi [26] discussed the changes in healthcare, public, government, energy, environment and industries in a parallel manner. However, in the current phase, the holistic and coupling impacts that focus on cross-sections have been rarely investigated. In addition, most of the existing observations are somewhat inconsistent across communities, countries and regions, which not only cannot fully describe the impacts, drivers and complexity of the COVID-19 pandemic but which disorient public opinion on the impacts [27]. As COVID-19 has been developing, and now even escalating fast, the previous overviews and reviews are becoming outdated very fast. There are always different angles of view and various observations, as implied by the parable of the blind men and the elephant in Figure 1. The novel contributions of this paper are highlighted as follows:

- The structural changes in healthcare, energy and environment, in particular the cross-sections of the three sectors, have been overviewed, which offers insights and perspectives for the in-depth understanding of the complicated COVID-19 impacts. Through the broad overview, several interesting and practical observations have been highlighted.
- A novel concept of the healthcare–energy–environment nexus under climate change constraints has been proposed and discussed to potentially guide more balance and innovation about the sustainable development of the healthcare–energy–environment coupling system.
- Based on the proposed nexus, the dynamics of the attention, being triggered by COVID-19, to healthcare, energy and environment in view of decision-makers have been illustrated, which is taken to analyse the on-going situation and look ahead future scenarios to sustainable development goals.

The paper contains another five sections. Section 2 overviews the changes in healthcare systems from energy and environmental perspectives. Section 3 overviews the changes in energy consumption and its relevance to healthcare and the environment. Section 4 overviews the changes in the environment related to air pollution and solid waste. Section 5 illustrates the healthcare-energy-environment nexus, attention dynamics and future outlook. Section 6 concludes this paper.

## 2. The Changes in Healthcare Systems—Energy and Environmental Perspectives

COVID-19 is a disaster for healthcare systems. This section overviews and analyses the COVID-19 impacts on healthcare resources and other infectious and non-communicable diseases. Their relationships with energy and environmental footprints are also discussed.

### 2.1. Impacts on Healthcare Resources

During the COVID-19 pandemic, most global healthcare resources, such as ventilators, protective equipment, healthcare workers and healthcare-related energy supply [28], were allocated to the prevention, control and treatment of coronavirus infection [29]. This is the case, especially in the epicentres of the disease outbreak in different countries, which led to the insufficiency of healthcare resources [30] and a great burden of healthcare systems [31]. The fair allocation of limited healthcare resources was urgently appealed [32], especially for African countries [33]. A depletion in medical resources would result in both increased COVID-19 mortality and elevated all-cause mortality [34]. The challenges in healthcare resources influence mitigation strategies and increase the energy and environmental footprints:

- The severe shortage of medical resources such as hospital beds, ventilators and intensive care unit (ICU) beds would destroy the crisis management unless the pandemic curve of COVID-19 cases could be flattened over a long time [32] by containment measures or vaccines support. However, several years are needed to produce a licensed vaccine, even at the pandemic speed [35]. To this end, the lockdowns were broadly implemented in worldwide countries [36]. Although the lockdown strategy would, in turn, put pressure on the healthcare resources and the sustainability of the energy sector, it is a compromise by considering both the healthcare resources and the flatness of the infection curve.
- The energy and environmental footprints have been increased by the global emergent production and logistics of healthcare resources due to the resource shortage and the regional heterogeneity in healthcare resource accessibility and availability. For example, the energy consumption by emergency logistics is about 17 times that by regular logistics [37]. Before the COVID-19 crisis, the healthcare sector has been one of the major contributors to climate emissions. Healthcare sector’s footprint is 4.4% of global net greenhouse gas (GHG) emissions [38]. Healthcare activities in the middle- and low- income countries counted for 3 to 5% of national total carbon emissions, and the proportion in some developed countries ranged from 5 to 15% [39]. For example, the GHG emissions related to healthcare activities contributed 8% of total emissions in the US in 2007 [40], and this proportion was enlarged to 9.8% in 2013 [41]. Such proportions in both developed and developing countries would be understandably increased under the COVID-19 circumstances.

With the above lessons, at least four aspects of enhancement would benefit the current and future healthcare systems. First, in resource-limited settings, a quick energy-infrastructure gap assessment of existing facilities [7] could be helpful to select the most plausible solution of sustainable energy. Second, a reliable national public-health information support system [42] has been urgently needed to identify regional heterogeneity in the demand of direct healthcare resources in different areas. Third, it is vital to improving energy security and energy efficiency [43], especially in the healthcare sector. Fourth, since climate risks and COVID-19 risks have been compounded [44], healthcare and climate and environment issues should not be isolated.

### 2.2. Impacts on other Infectious Diseases

The co-epidemics of infectious diseases have overwhelmed the healthcare systems, for example, the co-epidemics of COVID-19 and dengue fever [45] and the co-epidemics of COVID-19 and flu [46]. As the multi-stress healthcare systems would further endanger energy and environmental sustainability, the in-depth understanding of the co-epidemics is an urgent and key task.

Dengue, a global infectious disease in more than 100 countries, caused about 390 million infections annually [47]. During COVID-19, severe outbreaks of dengue fever were observed in South and South-East Asia [45] and Latin America [48] where a huge part of the world population lives. Before mid-August 2020, the annual dengue incidence reached historical records in Paraguay [49] and Singapore [50]. There is quantitative evidence implying the potential relationship between the lockdown and the excessive number of dengue cases. During the two-month lockdown period in Singapore, compared to the previous two months, “a five-fold increase in the incidence of Aedes mosquito larvae detected in homes and common corridors in residential areas” [51]. Regarding the excessive number of dengue cases in 2020, there is another possibility that more dengue fever patients with mild symptoms were tested due to the COVID-19 outbreak. The primary care providers were suggested to test patients with fever even without a clear COVID-19 source and make sure earlier identification [52], partially since COVID-19 and dengue fever present similar laboratory and clinical features [53]. The impacts of lockdown and resulting impacts on the dengue infection and control needs more investigation based on big urban data of the dengue transmission dynamics and the pattern changes of population mobility. Another interesting evidence showed less COVID-19 transmission and infection were observed in highly dengue-endemic countries, including India, Bangladesh, Singapore, Malaysia, Japan, Mexico, Brazil, Argentina and Sudan [54], which was later enhanced by the new data from Brazil [55]. However, such observation seemed not to be confirmed by the Singapore case. Spatial-temporal data analytics are important to draw or update a conclusion about such public health issues to avoid bias or misleading. In addition, other underlying common factors that may influence both the COVID-19 and dengue transmission deserve further investigation from a causality perspective rather than a relationship perspective to promote an in-depth understanding.

Worldwide flu transmission seemed to be lessened by containment measures according to global samples from 71 countries [56]. Typical cities and countries include New York, NY, USA [56], Singapore [57], Japan, Qatar and several countries in the southern hemisphere [46]. However, there is another possibility that patients with flu would rather (or have to) stay at home since COVID-19 has overwhelmed the medical systems [46]. The next flu season is approaching in the northern hemisphere. It is better not to take the mildness of flu as for granted, let alone many countries have lifted lockdowns to recover the economy, and some people have been tired of wearing masks and even angered of staying-at-home [58]. Similar concerns have been reported for other infectious diseases, e.g., tuberculosis. During the pandemic, a drop in the number of tuberculosis patients visiting hospitals or clinics has been reported by at least 121 countries [59]. Lockdowns and the resulting disruptions in supply chains and medical services hindered the progress of about 80% of tuberculosis, human immunodeficiency virus (HIV) and malaria programs [59]. The projection data indicated that an additional 6.3 million cases and 1.4 million global deaths in the next five years could be caused by a worst-case situation of global lockdowns [60]. This could be regarded as potential energy and environmental footprints due to lockdown measures. Prioritising COVID-19 and its healthcare over energy and environment is somewhat understandable during the crisis. However, it is not so rational to always prioritise COVID-19 over other infectious diseases. More studies on identifying the optimal prioritising of diseases treatment, e.g., by little’s law [61], at different phases of the pandemic, are important to prevent the overlook of other pressing health issues.

It is high time to rethink about the lockdown and its implementation and exit manner. Some strategies have the potential to improve the implementation and exit of lockdowns, such as the intelligent quarantine strategy [62], the responsible lockdown exit strategy [63] and the flexible local lockdown exit strategy [64]. Although the function of lockdown is well recognised from the perspective of COVID-19 mitigation, the non-flexible lockdown causes disorder to healthcare systems with the incorporation of other infectious diseases threatens the global energy and environmental sustainability.

### 2.3. Impacts on Non-Communicable Diseases and e-Healthcare

According to an online survey with 202 healthcare professionals from 47 countries [29], the first two most impacted non-communicable diseases are diabetes and chronic obstructive pulmonary disease. Patients with non-communicable diseases, especially chronic diseases, have been enduring great pressures from the inconvenience of medical treatment and the higher risk of death after potential coronavirus infection. For example, patients with Type 1 or Type 2 diabetes are at risk of severe COVID-19 and generally have worse outcomes [65]. Elective medical services have also been cancelled around the world in combating COVID-19 [66]. In addition to physical health, another particular impact is that on mental health, especially to those with chronic diseases. The problem is brewing currently in all societies [67], and it will only get worse unless proactive recognition and offer of support are provided by the healthcare systems and governments. By the online survey, 80% of the healthcare professionals claimed deteriorating mental health of their patients during the COVID-19 pandemic [29].

Although telemedicine and e-healthcare technologies have been around as concepts in medical applications for some time already. Those have been the case non-communicable diseases mainly; however, COVID-19 understandably highlighted the importance [68] and boosted their rapid development [69] to reduce face-to-face contacts [29] via virtual healthcare, mobile devices, smart apps and digital technologies. Telemedicine and e-healthcare can be as very beneficial solutions during the COVID-19 period [5]. However, there are still pitfalls to deal with [70], e.g., medico-legal issues, in telemedicine consultation, which required further improvement and adaption. For COVID-19 fighting with digital technology, Ting et al. [71] summarised the related technologies, including the artificial intelligence (AI) which employs machine learning, big-data analytics, blockchain platform technology [72] and Internet of Things (IoT) with advanced telecommunication technology, e.g., 5G and even 6G. The development of telemedicine, e-healthcare and healthcare robotics has been promising [73]. A compound annual growth rate of over 33% has been expected in the global remote healthcare market from years 2019 to 2025 [74]. However, the online services and smart monitoring are generally less accessible for some populations, including the patients who live in low- and middle-income countries [75], the older patients with lower socio-economic status and less education, and even some patients without insurance coverage for telemedicine [76]. It requires substantial work to reach these individuals and eliminate payment barriers so that healthcare with digital technology can cover all of the target populations.

Another aspect that it is easy to overlook lies in energy and environmental footprints of telemedicine and e-healthcare. The chronic disease management in the data era is a multidisciplinary systems science, which connects people, data, devices and multiple systems [77]. In the year 2019, the global real-time virtual healthcare took over 46% of the overall e-healthcare market [74]. Massive data samples are needed to discover hidden patterns and even recommend better solutions [78], for which personal real-time monitoring is a general pathway [79]. Although telemedicine and e-healthcare would reduce the travelling of patients, the real-time monitoring, operations of smart devices, data storage, big-data analytics by AI consumed massive energy and produced unexpected air emissions. For example, the AI model training would produce the carbon emissions equivalent of five times that by the American cars [80]. The information and communication technology (ICT) sector produces 2% of the global carbon dioxide (CO_2_) emissions [81]. All digital technologies rely on data centres, and the energy-use related to only data centres accounted for ~1% of global electricity use [82]. During and after the COVID-19 pandemic, it requires more comprehensive assessments about the potential impacts of telemedicine and e-healthcare on energy consumption and GHG emissions, rather than just the assessment of the travelling-distance reduction [83] and its resulting benefits for global emissions. In addition, the monitoring quality and warning mechanism need to be improved to avoid unnecessary healthcare visits. For example, the abnormal pulse feature of Apple Watch would lead to excessive use of limited healthcare resources [84] and therefore increase energy and environmental footprints. The improvements in terms of AI algorithms and energy efficiency have the potential to reduce electricity consumption for long-term monitoring and data analytics [85]. Practitioners have been recently trying the energy-efficient IoT-health monitoring system [86] and the energy-efficient and secure framework based on Internet of Medical Things (IoMT) [87].

## 3. The Changes in Energy Consumption—Healthcare and Environmental Relevance

COVID-19 had also changed people’s social and economic activity patterns which had been reflected in products consumption and purchase [88], and energy consumption [89]. After implementing lockdowns in worldwide cities, the global energy demand and consumption dropped significantly, and apparent structural changes were observed. Although renewable energy demand is relatively stable, mainstream fossil energy demand drops [90]. The residential demand increases, but industrial and commercial demands drop [91]. According to the data from the International Energy Agency, the year-on-year demand growth rates of global oil, coal, gas, nuclear and renewables in 2020 compared with 2019 were estimated as –9.12, –7.73, –4.99, –2.52 and 0.79% [92]. The residential energy demand increases, but the industrial and commercial energy demands decline sharply due to the shutdowns [24]. For example, electricity consumption declined by <10%, whilst the consumptions of gasoline and jet fuel were reduced by 30 and 50% in the US [24]. In the energy sector, too much attention has been paid on declined energy consumption. The extra energy consumption for COVID-19 fighting in the healthcare sector has been considered as a major issue. The actual decline of energy consumption was partly offset by extra energy consumption for COVID-19 fighting and was really declining during the spread-out lockdown. Klemeš et al. [37] appealed to focus more on the extra energy footprints and extra environmental footprints of those fighting measures during COVID-19, e.g., personal protective equipment, disinfection and supply chains even if energy and environmental issues understandably receive relatively lower priorities compared with healthcare issues in the critical situation. In addition, the potential rebound effect in overall energy consumption of digitalisation and IoT, which was expected to be significantly stimulated by COVID-19 crisis, needs to be further assessed. Most of the studies suggested that it is rather optimistic that digitalisation could lead to lower energy consumption. IoT and AI could lead to higher energy efficiency; however, the increase in the energy usage of IoT and AI could outweigh the positive impacts [93].

Under current technology conditions, the development in the energy sector would influence environmental benefits [94]. However, the energy sector also provides electricity support for environmental management. In the opposite direction, the environmental sector offers resources for the energy sector, which constitutes a natural nexus between them. The COVID-19 has navigated the transitions of renewable and sustainable energy [95] and clean energy, including nuclear energy [96]. According to data from 123 countries over 25 years, Sovacool et al. [97] found that, compared to renewables, nationwide nuclear attachments do not necessarily associate with lower carbon emissions significantly. More attention and personalised assessment, with a consideration of the environment in different countries, are urgently required to navigate the energy transitions under the COVID-19 pandemic.

The energy sector provided strong support for healthcare during COVID-19. Klemeš et al. [37] summarised non-negligible energy and environmental footprints in terms of hospitalisation, personal protective equipment, working shift, food packaging, disinfectants, massive testing and supply chains in the healthcare systems. The measures for COVID-19 mitigation escalate energy demand to address the issues in the healthcare sector [94]. When the two topics—namely, energy and healthcare—meet, the energy reliability and energy justice cannot be bypassed:

- With sufficient collaboration between the healthcare and energy sectors, healthcare facilities and services rely on reliable electricity [28] and affordable energy, especially the clean and renewable power [98] to effectively treat patients. The reliable energy used for COVID-19 response was regarded as one of the main missing links in the healthcare systems of underdeveloped countries [7]. For connecting such a missing link, Rinkoo et al. [7] recommended a “green public-health infrastructure concept” for the COVID-19 response.
- The COVID-19 pandemic is not only a health and economic crisis but also a justice crisis [99]. Generally, as the rural areas lack access to electricity and 24/7 health services, distributed energy systems with local clean and renewable sources might be an appropriate solution for healthcare centres in such areas [98]. The development of both energy and healthcare technologies would contribute a breakthrough to the justice issue.

## 4. The Changes in the Environment—Air Pollution and Solid Waste

Section 2 and Section 3 have discussed the relationships amongst healthcare, energy and environment. Section 4 provides a critical overview of the impacts of COVID-19 on global air pollution and solid waste. Most previous studies emphasised that (i) air quality has been improved due to global lockdowns with the priority in the healthcare sector and (ii) solid waste generation was observed to be reduced significantly in worldwide cities. However, this section presents some systematic thinking to eliminate such biased optimism. Each paper has its own focus. This study mainly concentrates on spatial heterogeneity and potential adverse impacts on air pollution and solid waste. For more details of impacts of COVID-19 on the environment, including the wastewater [100], river water [101] and environmental noises [102], the readers are referred to the short review regarding COVID-19 and the environment [103] and the overview regarding the observed and potential impacts on the environment [104].

The forced confinement had decreased global CO_2_ emissions by 17% (11 to 25% for ±1σ) by early April 2020 compared with the average CO_2_ emissions in 2019 [105]. A temporary reduction in GHG emissions had been reported consecutively in worldwide cities during the period of implementing lockdowns. The spatial heterogeneity of the reduction rate of CO_2_ emissions in the first half-year of 2020 was calculated by Liu et al. [106], Spain (−18.8%), India (−15.4%), Germany (−15.1%), the United Kingdom (UK) (−15.0%), France (−14.2%), Italy (−13.7%), the US (−13.3%), Brazil (−12.0%), Japan (−7.5%), Russia (−5.3%) and China (−3.7%). In addition to the heterogeneity on the country scale, data from the 28 air quality stations in the US indicated that the reductions in nitrogen dioxide (NO_2_) ranged 5 to 49% [107], and different mechanisms on temperature, air pollution and COVID-19 cases have been identified by regional divisions based on the 219 prefecture cities in China [108]. On 9 September 2020, the United in Science 2020 Report showed that GHG concentrations continue to climb and have been at record levels in the atmosphere following a temporary decline because of COVID-19 [109]. With the recovery of population and economic activities, the long-run GHG emissions are still hard to estimate. For example, stimulus packages and actions taken by governments might lead to the dual growth of the economy and GHG emissions in the coming years [110]. Even in the short term, the situations are not necessarily all positive. Although sulfur dioxide (SO_2_) and NO_2_ were recorded at the lowest levels amongst the past six years during the lockdown period, extreme particulate matter concentrations were observed unexpectedly in northern China due to the synergy of multiple factors, including the stagnant airflow, the anomalously high humidity, the uninterrupted emissions from petrochemical facilities and power plants, and the alleviated titration effect during the lockdown [111].

Similar phenomena have been observed in Barcelona, Spain and Ontario, Canada during the lockdown period. Although the NO_2_ and black carbon were reduced significantly, the particulate matter concentrations had no apparent changes in Barcelona [112] and Ontario [113], and the concentration of ozone (O_3_) in the air in Barcelona increased by about 50% [112]. In addition, due to the lockdown and population’s indoor activities, the evidence from Spain indicated that the indoor air quality gets worse, where the mean daily concentration of 2.5 μm particulate matter increased by 12% and that of total volatile organic compound increased by 37 to 559% [14]. Although it was thought as a “Blessing in Disguise” at the early time of pandemics [114], it is challenging to make a straight conclusion on the impact of COVID-19 on air pollution from both the long-term and short-term perspectives. More in-depth analysis and understanding are urgently required.

**Figure 3:**
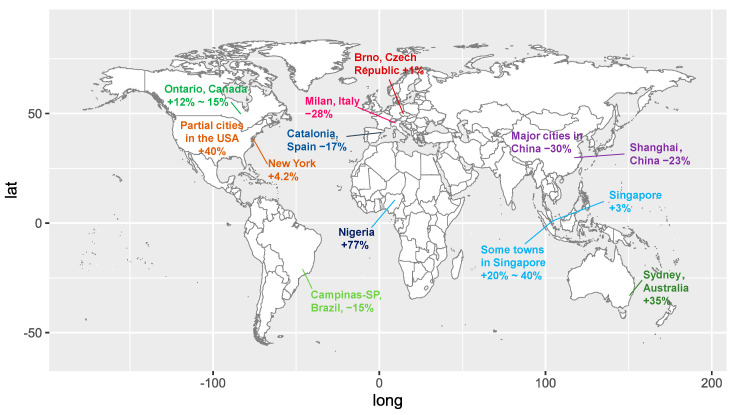
The spatial heterogeneity of the change rate of solid waste generation caused by COVID-19.

Significant structural changes in the solid waste generation were also observed apparently. Just in China, the production of face masks with plastic components increased to 116 million/d in February 2020, being equivalent to 12 times the amount in the previous month [115]. The massive usage of plastic in personal protective equipment boosts medical waste drastically, e.g., a whopping increase of 370% in Hubei, China [89] and 350% in Catalonia, Spain [116]. The lifestyle changes and the cost incentive of plastic production increase the overall plastic demand and the resulting plastic waste [117]. For example, a 40% increase in plastics demand has been expected in Spain, and a 15% elevation of plastic waste demand has been recorded in Thailand [116]. Plastic waste elevated from 1500 to 6300 t/d in Thailand at the peak time [118] owing to food deliveries to homes. What makes the situation worse lies in the destroyed plastic recycling programmes. For instance, over 40% of plastic recycling companies in South and Southeast Asia were at risk of bankruptcy due to the COVID-19 pandemic [119]. Figure 3 shows the spatial difference of the change rate of solid waste generation caused by COVID-19. Compared to medical waste and plastic waste, household waste has presented more complicated changes, e.g., a 28% reduction in Milan, Italy, and a 17% fall in Catalonia, Spain [116], a 25% reduction in Barcelona, Spain [120], a 15% fall in Campinas-SP, Brazil [121], a 23% decrease in Shanghai, China [122], and a 30% reduction in major cities of China [89].

However, due to the geographical, sociological and cultural factors, some cities present converse trends, e.g., a 1% elevation in Brno, Czech Republic [122], a 3% increase in Singapore [123], a 4.2% climbing in New York, US [124], a 12 to 15% increase in residential garbage in some municipalities of Ontario, Canada [125], and a 35% bloom in Sydney, Australia [126]. Some towns observed 20 to 40% more waste during the lockdown period in Singapore [127]. Waste in residential areas had spiked by as much as 40% in some cities in the US [128]. The household waste generation in Nigeria, Africa was roughly estimated to increase by 77% based on the questionnaires [129]. Systems thinking [130] and diversified solutions are needed for different regions and countries, and for different types of waste. It is notable that double whammies have been caused by the variation in solid waste generation. On the one hand, the treatment capacity in some densely populated cities would be the limitations for those sharply increased medical waste [89]. For example, over 35% of medical waste was not treated properly in Brazil [121]. Mismanagement of excess waste could result in increased environmental pollution [131] and even health risks due to medical waste pollution. On the other hand, although zero-waste under a Circular Economy paradigm would be a long-run trend, sharply varied amount of municipal waste might destroy the sustainability of operations of the incineration facilities in the waste-to-energy systems. Worldwide best practices during COVID-19 would be beneficial for emergency and disaster waste management.

In addition to the variation in waste generation, the behaviour changes in recycling and illegal waste dumping may bring long-term impacts on waste management. Recycling programmes might be hindered by several reasons, such as the resource shortage and the high risk of manual waste segregation during pandemics. For example, the government authority recommended the suspension of such programmes in Brazil due to the potentially high risks in recycling centres [121]. The medium- or long-term effects of the suspension of recycling programmes deserve concerns [132]. However, compared to the Brazil case, waste recycling also presents geographic differences. For example, the waste recycling rate in Milan, Italy, was increased by 1% compared with the same period in 2019 [120]. Shanghai is another positive example. Although the absolute volumes of household waste and recyclables were reduced, the recycling rate had no significant variation in Shanghai [133], where waste segregation at the source was implemented [134]. The lesson learned from the COVID-19 could (i) boost the automation-enhanced waste segregation using intelligent robotics [135], and AI technologies [136] and (ii) promote the sorting at the source [134] rather than at the end. During the COVID-19 pandemic, illegal waste dumping, including the face masks, was broadly observed in Brazil [121], the UK [137] and Romania [138]. For example, a 300% rise in illegal waste disposal was observed during the lockdown in the UK [137], and a 70% increase was reported in Melbourne, Australia in April 2020 [126]. As improper management of solid waste would increase the potential spread of COVID-19 [139], the illegal waste dumping could act as a risk factor to accelerate the spread of coronavirus and threaten the healthcare system.

## 5. Nexus, Attention Dynamics and Future Outlook

A novel concept— namely, a healthcare–energy–environment nexus—is introduced, which is followed by the discussion of dynamics of the attention to healthcare, energy and environment in view of decision-makers. With the concept and attention dynamics, the future outlook is outlined subsequently.

### 5.1. Healthcare–Energy–Environment Nexus

**Figure 4:**
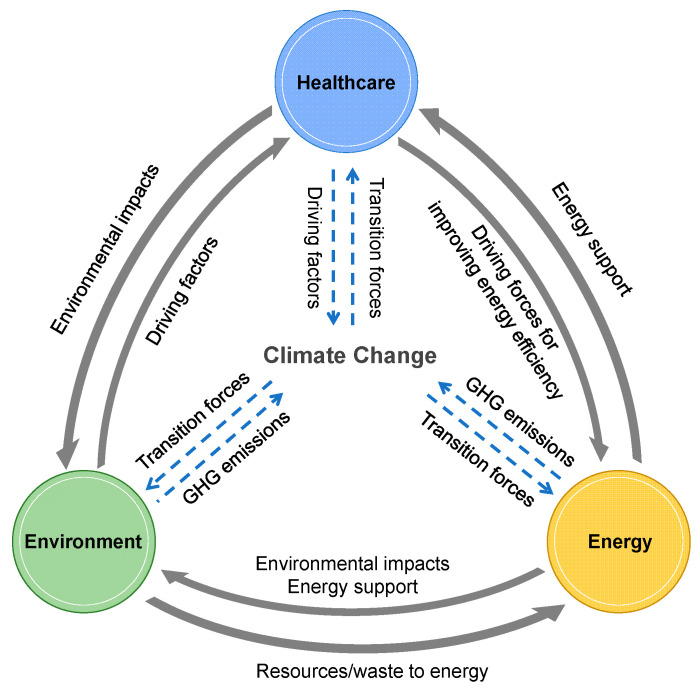
The conceptual diagram of the healthcare–energy–environment nexus under climate change constraints.

Through reviewing the COVID-19 impacts on healthcare, energy and environment, we propose a novel concept of a healthcare–energy–environment nexus under climate change constraints by referring to other nexus-related studies, such as the energy–climate–health nexus [140], food–energy–water nexus [141] and water–energy–GHG nexus [142]. As illustrated in Figure 4, the solid arrows suggest the direct relationships amongst the healthcare, energy and environment. Healthcare would cause environmental impacts and act as the driving factor for improving energy efficiency. The environment provides resources and waste to generate energy and acts as the driving factor for health issues in the healthcare sector. The energy sector provides necessary energy and electricity support to the healthcare and environment sectors and causes adverse environmental impacts as well. The dashed arrows in Figure 4 imply that healthcare, energy and environment trigger climate change that has been one of rethinking focuses during and after COVID-19 [143]. The fossil energy usage, air emissions, waste treatment by incineration, and healthcare with energy support and environmental impacts are all driving factors for climate change. Reversely, the proper regulations on climate change offer constraints to force the transitions of the healthcare, energy and environment management, e.g., the IoMT-based energy-efficient e-healthcare [87], the sustainable waste management under Circular Economy [144] and the transitions of clean and renewable energy [145].

The objective of healthcare is to save lives and improve human health. If less attention was focused on the relationships amongst healthcare, energy and environment, the healthcare itself might still function well but at the expense of climate footprint, which, in turn, has counterproductive effects to human health. With the healthcare–energy–environment nexus under climate change constraints, more balance and innovation can be potentially guided, which triggers the rethinking, redesign and rerun of the healthcare systems, especially the sustainable development of telemedicine and e-healthcare, in a healthy and sustainable society.

### 5.2. Dynamics of the Attention to Healthcare, Energy and Environment

**Figure 5:**
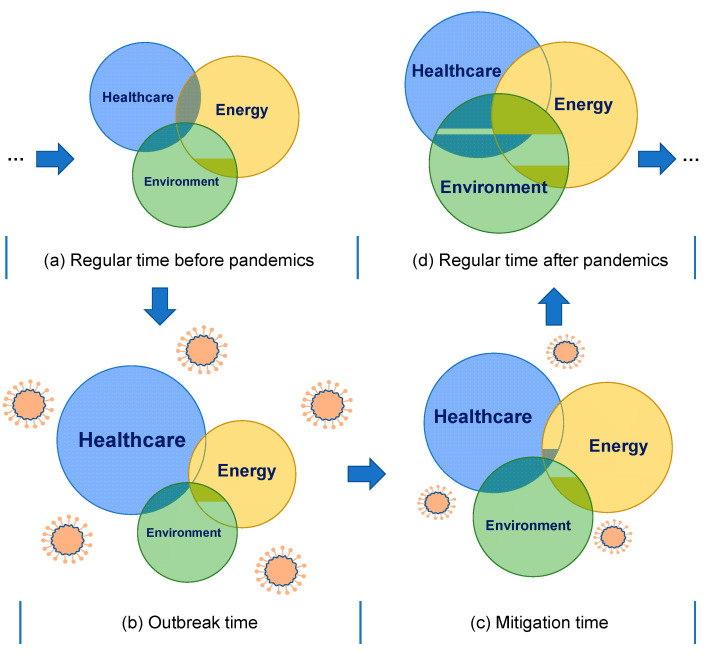
The dynamics of the attention to healthcare, energy and environment triggered by COVID-19. Note: The discussed attention in this study is in view of decision-makers. In Figure 5a–c, the circle size denotes the paid attention qualitatively, and the overlapping areas in the figure denote the relative attention for issues in the cross-sections. The diagram in Figure 5d is presented with some projection and expectation for the future scenario.

After a crisis, the priority of the attention to healthcare, energy and environment varies significantly. Figure 5 qualitatively shows the dynamics of the attention to the three sectors before, during and after (expected) the pandemic. According to the nexus in Figure 4, the attention also has overlaps. The dynamics are explained as follows:

(a) Before COVID-19, the promising development of healthcare, energy and environment (Figure 5a): The harmonious development has been receiving initial attention worldwide. The net zero-emissions healthcare by 2050 [38] is a good example to present the focus on healthcare’s climate footprint and its related factors. According to a survey with 3500 consumers [146], healthcare, energy and environment were thought to be the top three priorities for innovation and technology. Advanced technologies promoting the development of healthcare, energy and environment have been reviewed and commented by Lim et al. [147].
(b) During the COVID-19 outbreak, the emergency development of healthcare (Figure 5b): The COVID-19 pandemic not only prioritises the healthcare sector but also reduces the attention to the cross-sections (i.e., healthcare and energy, healthcare and environment and even energy and environment). The rapid response of COVID-19 fighting was seriously treated during the outbreak time, even if at the expense of energy waste and environmental pollution [37].
(c) During the mitigation time, healthcare still dominants energy and environment (Figure 5c): Compared to the outbreak time, the energy sector in the mitigation time has been paid more attention as it is directly related to the economic recovery. More environmental issues have been exposed in the mitigation time; the environment has been gradually back to managerial and public view. For example, both the energy and environment dimensions have been considered in the transition of sustainable supply and production during COVID-19 [148]. The ‘15-min city’ [149] has been appealed again to build a lifestyle with a safe and low-carbon environment, although the ‘15-min city’ is not a new concept.
(d) After the pandemic (projected), the repercussion and sustainable development with more balance and cross-section attention (Figure 5d): For the situation after the pandemic, it is projected that more attention might be focused on the cross-sections of healthcare, energy and environment, as illustrated in Figure 5d, which is discussed in the future outlook part in Section 5.3.

The conceptual diagram of dynamics in Figure 5 is simplified without considering the second-wave outbreaks [150]. However, the diagram does not lose its generality by adding more phases that adjust Figure 5b,c slightly, even if multiple-wave outbreaks occur.

### 5.3. Future Outlook and Suggestions

To approach multiple long-term goals in the healthcare, energy and environment sectors, such as the net zero-emissions healthcare by 2050 [38], the 100% renewable energy system by 2050 [151], the long-term “zero-waste cities” plan [152], and the climate-neutral by 2050 [153] in the heart of the European Green Deal [154], it would be beneficial to pay more attention to the cross-sections amongst healthcare, energy and environment, as shown in Figure 5d. The healthcare, energy and environment systems should be treated as an integrated objective to implement improvements synergistically. With the help of the healthcare–energy–environment nexus in Figure 4, quantitative assessments are urgently needed for the relationships amongst healthcare, energy and environment. Almost two-thirds of the 169 targets in the Sustainable Development Goals are now unlikely to be met [2], partially due to the COVID-19 pandemic. The healthcare–energy–environment nexus, as discussed in Section 5.1, might have the potential to assist in improving the related implementation strategies to original sustainable development goals.

Currently, the whole society has no absolute confidence that the COVID-19 would be wiped out even when we have vaccines. According to the study from a Harvard team, a pandemic may resurge in 2025, even if it is apparently eliminated [155]. In the worst scenario, the coronavirus may accompany human beings forever [156]. The defined regular time after pandemics in Figure 5d could just be a vision. In an extreme situation, the society would live with coronavirus wisely and may try to conduct the plan in Figure 5d during the mitigation time period. During COVID-19, the influence of coronavirus is not always more severe than those of other diseases. For example, the flu and pneumonia deaths were recorded three times coronavirus deaths for the week ending on 10 July 2020 [157]. COVID-19 might be a long-term fight. Based on such an assumption, the society should not mainly focus on COVID-19 but need to take care of other infectious and non-communicable diseases, energy and environment during the mitigation time period.

It is understandable that the impacts of the on-going COVID-19 pandemic may surpass those of historical pandemics. For example, the estimated deaths in New York City during the COVID-19 is more than 1918 influenza pandemics [158]. It is hard to imagine what would happen if the global COVID-19 pandemic had happened before. Although the world was at the atmosphere of panic, sadness, anger and anxiety due to COVID-19 [58], the new day’s sun has been rising, gratifying that we are already in the advancing stage of an era of digitalisation. There are many digital solutions for many problems, such as e-tracking for COVID-19 [62], telemedicine [69], e-healthcare [87], e-learning [159], e-office [160] and e-shopping [161]. As discussed in Section 2 to Section 4, the digitalisation and ICT, with proper support from design, production and manufacturing, have the potential to play the crucial role in coping with the pandemics, sustainable environment management and energy efficiency improvement. However, without proper design, the digitalisation is not necessarily a driver for sustainability [162]. Although the comprehensive assessment of the rebound effects of digitalisation and ICT is challenging [163], the energy cost [164], the environmental effect [163] and climate footprint [165] of digitalisation and ICT should not be neglected. Based on the proposed healthcare–energy–environment nexus, the future development framework of a climate footprint-focused digitalisation would be expected. More definitions and explanations of footprint analysis are referred to as Čuček et al. [166]. For such a potential framework, the climate change emergency [167], the Circular Economy strategies [168] and the transitions to renewable and sustainable energy [95] would make contributions from different perspectives. Beyond fundamental technical solutions, operational optimisation [169] and global collaboration and data sharing [170] have always been being pursued.

## 6. Conclusions

In the early stage of the COVID-19 pandemic, each piece of practical information contributes to understanding COVID-19 impacts on healthcare, energy and environment. Back to the ancient Hindoo Fable at the beginning of this paper, the fragmented understanding lays the foundation for systematic integration. In the mitigation time period, it is urgent to know more about the comprehensive impacts to guide us to move ahead. This study has been discussing the relations amongst healthcare, energy and environment during the early stages, the rising period of the pandemics and the projected post-pandemic period. The main stress was to a deeper understanding of those consequences of contributing to curtail various negative impacts. The point by point observations are summarised as follows:

- During COVID-19, healthcare has been prioritised at the expense of energy and environmental costs. The extra energy consumption and extra environmental footprints, due to the blooms of e-healthcare and the regional heterogeneity in healthcare resource accessibility and availability, deserve more assessment.
- The lockdown measures due to COVID-19 do not necessarily benefit other infectious diseases, which may also cause extra energy and environmental footprints in the following years. The weight of priority for different infectious diseases should be adjusted dynamically, and smarter and more flexible lockdown strategies are worthy of more investigation.
- The energy sector plays a strong support role in effective healthcare and environmental management. COVID-19 has speeded up the energy transitions, for which more attention and personalised assessment with a consideration of the environmental footprint in different countries are needed to navigate the energy transitions under the COVID-19 pandemic.
- Although GHG emissions have been reduced temporarily by global lockdowns, spatial differences are significant in the reduction rate, varying from 3.7 to 18.8%. More particulate matter and O_3_ pollution have been observed in several regions. The long-run GHG emissions and air pollution are still hard to estimate accurately due to the recovery of economic activities and stimulus packages during and after the pandemic.
- After implementing containment measures, the change rate of solid waste generation presents spatial heterogeneity in worldwide cities with the change rate ranging from –30 to +40%. The sharply varied and uncertain fluctuations in the amount of municipal solid waste might severely threaten the sustainability of operations of the incineration facilities in the waste-to-energy systems.
- Although industrial plastic recycling has been hindered, household waste recycling programmes are not necessarily affected significantly by COVID-19. The recycling programmes with waste segregation at the source have a slight impact; whilst those with waste segregation at the end have been affected greatly. The phenomenon of illegal waste dumping was observed more frequently during COVID-19, which may accelerate the spread of the virus and threaten the healthcare system.
- In spite of in its early stages, digitalisation plays an important role during this pandemic, e.g., e-healthcare, energy digitalisation and digital waste management. A climate footprint-focused digitalisation development pathway under the incorporation of the Circular Economy and the transitions of clean and renewable energy has a great potential to take off.

Based on these observations and the relations amongst healthcare, energy and environment, a healthcare–energy–environment nexus under climate change constraints is proposed and discussed. This nexus thinking, proposed in this study, could potentially guide more balance and innovation about the sustainable development of the healthcare, energy and environment sectors. As knowledge accumulates, information with insightful observations in cognitive blind spots helps the society illustrate the whole episode of COVID-19 impacts, which also triggers rethinking and redesigning pandemic mitigation measures. We are not sure whether the earth will encounter a ‘Disease X’ [171] in the future, but with the experience learned from this global crisis, the researchers hand in hand with the other professions and mainly politicians shall by strongly engaged for the society to be better prepared for that if it really comes.

In the future, some further works and possible directions are of worth to be explored. First, the healthcare–energy–environment nexus deserves more in-depth investigation in striking the right balance between the welfare of human beings (healthcare and energy) and planet (the environment), in other words, planetary health. Second, more quantitative assessments are also needed for the cross-sections of the three sectors, such as the energy footprints in the healthcare sector and the quantitative assessment of GHG emissions under the nexus thinking for healthcare, energy and environment. Third, multiple sectors, e.g., economy and ecology, might be incorporated into the current healthcare–energy–environment nexus to provide more insights. Fourth, COVID-19 has created many opportunities regarding large-scale natural or behavioural experiments for research. For example, a rapid learning experiment regarding effective strategies for climate change [172]. More studies in the healthcare, energy and environment sectors may focus on the lessons and structural changes related to COVID-19 [173]. Fifth, the strength of this review paper is the comprehensive discussion on the structural changes and spatial heterogeneity, while the possible limitation lies in the lack of enough critical comments for some of the findings and related reasons. The limitation could be addressed in the future when the knowledge on the impacts of COVID-19 is getting mature.

## Data Availability

The data used in this study are from open-source pathways, which have been correctly cited.
